# Development of a Novel Chocolate Utilizing Mushroom Fermentation and Associated Changes in Beneficial Components

**DOI:** 10.3390/foods15061045

**Published:** 2026-03-16

**Authors:** Shiori Fukuda, Momoka Nakata, Yuka Sameshima, Naomi Takemoto, Tokumitsu Matsui

**Affiliations:** 1 Department of Food Sciences and Nutrition, Graduate School of Food Sciences and Nutrition, Mukogawa Women’s University, Nishinomiya 663-8558, Hyogo, Japan; sfuku@mukogawa-u.ac.jp; 2 Department of Food Science and Nutrition, School of Food Science and Nutrition, Mukogawa Women’s University, Nishinomiya 663-8558, Hyogo, Japan; 2111276@mwu.jp; 3 Department of Food and Nutrition, Faculty of Human Life Studies, Hagoromo University of International Studies, Sakai 592-8344, Osaka, Japan; ysameshima@hagoromo.ac.jp; 4 Department of Health and Nutrition, Faculty of Food Culture, Baika Women’s University, Ibaraki 567-8578, Osaka, Japan; n-takemoto@baika.ac.jp

**Keywords:** mushroom, fermented foods, cacao, chocolate, theobromine, caffeine, polyphenol, antioxidant activity

## Abstract

This study investigated the secondary fermentation of cocoa beans using mushrooms to further improve the quality of beans. Cocoa beans were fermented using 42 species of basidiomycetes and ascomycetes. Mycelial growth was observed in 29 strains. When 75% cocoa chocolate was prepared using the cocoa beans in which mycelial growth was observed, theobromine concentration was higher in 17 strains compared with the control. Furthermore, caffeine concentration was similar to or lower than the control in all strains. Chocolate produced using cocoa beans fermented with particularly *Polyporus arcularius*, *Peziza vesiculosa*, and *Urnula craterium* exhibited significantly higher theobromine concentrations. Compared to the control theobromine concentration of 7.53 mg/g, *P. arcularius* showed 9.25 mg/g, 9.13 mg/g for *P. vesiculosa*, and 9.05 mg/g for *U. craterium*. Furthermore, the reducing sugar concentration and total polyphenol concentration increased, and the antioxidant activity was similar to or higher than that of the control. These results suggest that secondary fermentation using mushrooms could be used to develop chocolate characterized by high theobromine, low caffeine, and rich polyphenol content.

## 1. Introduction

Chocolate is a popular confection. It is primarily made from cocoa beans and cocoa butter, to which sugar, milk powder, and emulsifiers are often added in commercial products. Cacao beans are harvested as cacao pods in producing countries. After harvesting, the pods are split open, and the seeds, still attached to the cacao pulp, are collected. The seeds are fermented for about a week, then dried and shipped.

The fermentation methods primarily consist of the Heap method, where cocoa beans are piled outdoors and covered with banana leaves or sheets, and the Box method, where beans are placed in fermentation boxes for controlled processing. In both methods, the fermentation of the cocoa beans is affected by the microorganisms adhering to the surface of the cacao pods, the tools and facilities used, and the surrounding soil [1]. Regarding microorganisms, yeast, lactic acid bacteria, acetic acid bacteria, and spore-forming bacteria are commonly used [1,2]. In the early fermentation stage, yeast and lactic acid bacteria convert sugars in the cacao pulp into ethanol and lactic acid through alcohol fermentation and lactic acid fermentation, respectively. In the middle stage, acetic acid bacteria convert ethanol into acetic acid through acetic acid fermentation. In the late stage, many microorganisms die due to the accumulation of their own metabolites and rising temperatures, and spore-forming bacteria become dominant. These fermentation processes not only alter the components of the cacao bean but also induce biochemical changes within the bean due to the lowering of pH by fermentation products and the generation of fermentation heat. These changes in pH and temperature activate endoproteases and carboxypeptidases in the cacao beans, producing free amino acids and reducing sugars in the bean. These undergo the amino-carbonyl reaction and Strecker degradation during the roasting process, producing aromatic compounds that form the characteristic flavor of chocolate [1,2,3].

During fermentation, polyphenols, caffeine, theobromine, and other alkaloids in cacao beans are oxidized, polymerized, and dissolved, resulting in a decrease in their content [1]. Although fermentation of cacao beans plays an important role in making chocolate, the fermentation process is often performed by farmers who use naturally occurring microorganisms that are difficult to control.

Therefore, in recent years, methods have been reported to improve cocoa bean quality by introducing carefully selected lactic acid bacteria into harvested cocoa beans and fermenting them [4], or by controlling fermentation temperature [5]. Among these, research has been conducted on improving cocoa bean quality through re-fermentation of dried, unfermented cocoa beans [6] and secondary fermentation [7], it is considered possible that even cocoa beans that have undergone fermentation processing can have their chocolate quality further enhanced and develop unique flavors through additional fermentation.

Mushrooms are the name given to the fruiting bodies formed by fungi during sexual reproduction, which are sufficiently large to be clearly visible to the naked eye. Such fruiting bodies are formed by some Ascomycetes and many Basidiomycetes; these fungi are referred to as mushrooms [8,9]. The distinction between Ascomycetes and Basidiomycetes is primarily based on the characteristics of their sexual spores. Ascomycetes form sexual spores (ascospores) inside asci, whereas Basidiomycetes produce sexual spores (basidiospores) externally on basidia [10]. However, both possess various enzymes, including carbohydrate-degrading enzymes [11]. Consequently, applications utilizing mushroom enzymes in fermented foods have been reported, irrespective of whether the fungi belong to the Ascomycetes or Basidiomycetes [12].

For example, mushrooms possess various enzymes, including amylase, protease, alcohol dehydrogenase, and lactate dehydrogenase. These enzymes have been utilized for the development of miso and alcoholic beverages [13,14,15,16,17]. Fermentation using mushrooms increases antioxidant activity and produces new beneficial components. Previous studies have reported that post-fermented tea prepared using the wood-decaying fungus *Schizophyllum commune* showed a 36.5% increase in antioxidant activity compared to the control, and approximately double the concentration of total polyphenols and total catechins [18].

These findings suggest that fermented foods with superior antioxidant properties and beneficial components compared with conventional fermented foods can be developed by utilizing the fermentation capabilities of mushrooms. However, to date, no research has been conducted on fermenting cacao beans with mushrooms, and no chocolate made from mushroom-fermented cacao beans is currently being sold.

Cocoa beans are rich in polyphenols, and their usefulness is being researched, but the amount of polyphenols varies depending on the origin, type of bean, and fermentation period [19].

However, if it is possible to increase the amount of polyphenols by fermenting cacao beans with mushrooms, it will be possible to develop chocolate rich in polyphenols regardless of the origin of the cacao beans or the fermentation period, and the quality of chocolate can be expected to improve.

Furthermore, it is unclear how components such as theobromine and caffeine contained in cacao beans change due to fermentation with mushrooms. Clarifying this could help discover the value of chocolate produced by fermentation with mushrooms.

This study aimed to develop a new chocolate by fermenting cocoa beans using mushrooms. We conducted secondary fermentation of cocoa beans that had undergone fermentation processing at the producer farm using mushrooms and analyzed changes in the beneficial components within the cocoa beans.

## 2. Materials and Methods

### 2.1. Pre-Culture of Test Strains

The test strains (mushroom mycelium) consisted of 42 species of basidiomycetes and ascomycetes, including strains distributed by the National Institute of Technology and Evaluation’s Biological Resource Center (NBRC) and strains isolated by the authors. Plate media consisted of potato dextrose agar (Shimadzu Diagnostics Corporation, Kyoto, Japan), 2% malt extract agar (Nacalai Tesque, Inc., Kyoto, Japan), or Matsutake agar prepared to contain 0.5% glucose (Fujifilm Wako Pure Chemical Corporation, Osaka, Japan) and 2% yeast extract (Nacalai Tesque, Inc., Kyoto, Japan). For pre-cultivation, each mushroom mycelium was inoculated onto the appropriate plate medium recommended by NBRC and cultured at 25 °C.

### 2.2. Preparation of Fermented Cocoa Beans

Cocoa beans fermented in the country of origin (Ichinomiya Bussan Corporation, Osaka, Japan, Ghana origin) were autoclaved (121 °C, 20 min). After cooling under aseptic conditions, they were aseptically immersed in an autoclaved solution of 1% glucose and 0.5% peptone (Nihon Pharmaceutical Co., Ltd., Osaka, Japan) for at least 17 h.

The pre-cultured mushroom mycelium was inoculated onto fresh appropriate agar plates (5 mm squares × 10 plates). After soaking, 20 pieces of moisture-removed cacao beans were placed onto the agar plates inoculated with mushroom mycelium and cultured statically at 25 °C.

The end of cultivation was defined as the stage where mushroom mycelium covered the cocoa beans and grew over 80% of the entire Petri dish. For cultures where mushroom mycelium growth covering at least 80% of the Petri dish was not confirmed even after 100 days or more of incubation, the culture was terminated, and the preparation of a fermented chocolate was not performed. The control consisted of cacao beans autoclaved and then soaked in a 1% glucose and 0.5% peptone solution. The control and mushroom-fermented cocoa beans were each cultured in three separate Petri dishes.

### 2.3. Preparation of Fermented Chocolate

The preparation of chocolate from fermented cacao beans was based on the method reported by Kasai et al. [20], modified to suit this study. Fermented cocoa beans after static incubation were roasted in a household oven (110 °C, 40 min) while still covered with mushroom mycelium, then cooled overnight. After cooling, the mycelium and thin skins adhering to the cocoa beans were removed. The beans were then ground to a smooth consistency using a universal mill (Ichinomiya Bussan Corporation, Osaka, Japan). White superior soft sugar was added to achieve a 75% cocoa content, and chocolate was prepared. No other auxiliary ingredients were added.

### 2.4. Measurement of Theobromine and Caffeine Concentrations

Theobromine and caffeine concentrations were measured using high-performance liquid chromatography (HPLC). The HPLC system employed a DP-8020 pump (Tosoh Corporation, Tokyo, Japan) and a UV-8020 detector (Tosoh Corporation, Tokyo, Japan), with detection at UV 273 nm. The column used was TSKgel ODS-100V (Tosoh Corporation, Tokyo, Japan), and the mobile phase was a 30% methanol solution, with a flow rate of 1 mL/min. A sample preparation for theobromine and caffeine concentration analysis was performed according to the method reported by Saotome et al. [21]. Specifically, 0.1 g of fermented chocolate was mixed with 500 μL of 100 mmol/L carbonate buffer (pH 11) and 300 μL of hexane. The mixture was vortexed for 30 s, then heated at 50 °C for 10 min. After heating, 450 μL of 100 mmol/L carbonate buffer (pH 11) was added. The mixture was vortexed (30 s × 3 times), centrifuged (15,000 rpm, 20 °C, 10 min), and the hexane layer was removed. After hexane removal, 650 μL of 1 mol/L ammonium bicarbonate was added, the mixture was vortexed, and the supernatant was used as the test sample. The concentrations of theobromine and caffeine in each sample were determined using calibration curves prepared from their respective standards.

### 2.5. Measurement of Reducing Sugar Concentration

Reducing sugar concentration was measured using the Somogyi–Nelson method [22]. Specifically, an equal volume of distilled water was added to the fermented chocolate, the mixture was vortexed for 30 s, centrifuged (15,000 rpm, 4 °C, 10 min), and the resulting supernatant was used as the aqueous extraction sample. Somogyi’s copper reagent (Merck Ltd., Darmstadt, Germany) was added to 120 μL of the aqueous extract sample and heated at 100 °C for 10 min. After cooling on ice for 5 min, 120 μL of Nelson color reagent (Merck Ltd., Darmstadt, Germany) and 1.0 mL of distilled water were added. After standing for 15 min, measurements were taken at a detection wavelength of 500 nm using a microplate reader (Tecan Japan Co., Ltd., Kanagawa, Japan). The reducing sugar concentration of each sample was determined using a calibration curve prepared from glucose standards.

### 2.6. Measurement of Total Polyphenol Concentration

The total polyphenol concentration was measured using the Folin–Ciocalteu method and expressed as epicatechin equivalents [23]. Specifically, for defatting 0.2 g of fermented chocolate was mixed with 1.0 mL of hexane, heated at 50 °C for 10 min, centrifuged (15,000 rpm, 20 °C, 5 min). The hexane layer was removed, and the extraction was repeated under the same conditions. Residual hexane was then completely removed using a centrifugal evaporator (Tokyo Rikakikai Co., Ltd., Tokyo, Japan) to make the defatted sample. A sample extracted with 50% methanol from the defatted sample was used as the test sample. Epicatechin (Fujifilm Wako Pure Chemical Corporation, Osaka, Japan) was used as the standard substance, and absorbance measurements were performed at a detection wavelength of 765 nm using a microplate reader.

### 2.7. Measurement of Antioxidant Activity

Antioxidant activity was measured by chemiluminescence using a microplate reader according to the method of Tabata et al. [24]. Specifically, we measured the ability to scavenge superoxide (O2-) among oxygen radicals as antioxidant capacity. Each well was loaded with 10 μL of the water-extracted fermented chocolate sample, 85 μL of 0.1 M KH_2_PO_4_ buffer (containing 0.05 M EDTA·2Na, pH 7.5; hereafter referred to as KH_2_PO_4_ buffer), 30 μL of 100 μg/mL xanthine oxidase solution, and 5 μL of 45 mM MPEC (2-Methyl-6-p-methoxyphenylethynylimidazopyrazinone: ATTO CORPORATION, Tokyo, Japan) solution.

The microplate was loaded into a microplate reader, and 25 μL of 0.72 mM hypoxanthine (containing 0.05 mM EDTA) was dispensed using the built-in pump. The accumulated luminescence was then measured for 30 s. As a positive control, 10 µL of 0.1 M KH_2_PO_4_ buffer was placed in the well instead of the fermented chocolate sample, and the accumulated luminescence value was measured in the same manner. Antioxidant activity was calculated as: oxidation inhibition rate (%) = [(luminescence accumulation value of positive control − luminescence accumulation value of sample)/luminescence accumulation value of positive control] × 100.

### 2.8. Statistical Analysis

All results were obtained from three independent experiments. Statistical analyses were performed using IBM SPSS Statistics (version 26; IBM Japan Ltd., Tokyo, Japan) with one-way analysis of variance. When significant differences were detected, Dunnett’s multiple comparison test was applied for comparisons with the control group. Results are presented as mean ± standard deviation, and significance was defined as *p* < 0.05.

## 3. Results and Discussion

### 3.1. The State of Fermented Cacao Beans Using Mushrooms

**Figure 1:**
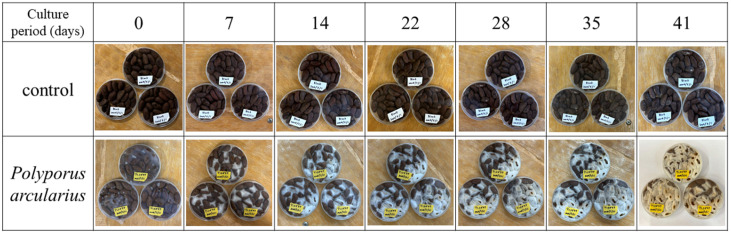
Appearance of cocoa beans during cultivation.

Figure 1 presents the appearance of cacao beans fermented with *Polyporus arcularius* as a representative example. Cultivation was terminated when mycelium growth covered over 80% of the entire Petri dish, as observed with *P. arcularius*.

Cocoa beans were fermented using 42 mushroom mycelium. Hyphal growth was observed in 29 mushroom mycelium and no hyphal growth in 13 even after a culture period exceeding 100 days (Table 1). The incubation period during which mycelial growth on cocoa beans was confirmed was 40 to 50 days for most mushrooms, with some strains showing growth even during shorter incubation periods of 20 to 30 days. No correlation was observed between the incubation period for mycelial growth and mushroom order, family, or genus.

This shows the appearance of cocoa beans during fermentation, comparing a control group not fermented with mushrooms and a group fermented using *P. arcularius*.

### 3.2. Theobromine and Caffeine Concentration

**Figure 2:**
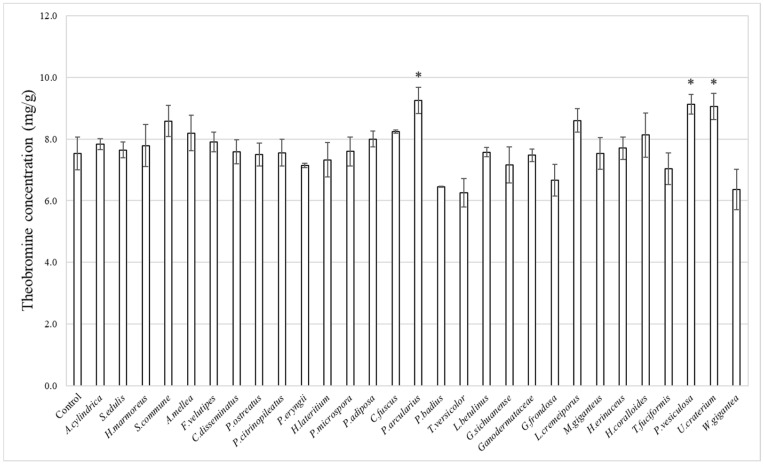
Theobromine concentration in fermented chocolate (mg/g). The error bars in the figure represent the standard deviation (*n* = 3). Asterisks (*) indicate significant differences compared with the control (*p* < 0.05).

Fermented chocolate was prepared using the 29 strains that exhibited mycelial growth. We found that the theobromine concentration in fermented chocolate exceeded the control in 17 strains, suggesting the potential for new theobromine production during fermentation (Figure 2). In the control group without mushroom fermentation, the theobromine concentration was 7.53 mg/g. However, in fermented chocolate using *P. arcularius*, it was 9.25 mg/g, *Peziza vesiculosa*-fermented chocolate showed 9.13 mg/g, and *Urnula craterium*-fermented chocolate showed 9.05 mg/g, indicating significantly higher theobromine concentrations.

*P. arcularius* is a basidiomycete belonging to the order *Polyporales*, family *Polyporaceae*, and genus *Polyporus badius*. However, chocolate fermented using *Polyporus badius*, a species within the same genus, exhibited lower theobromine concentrations compared with the control. Similarly to the cultivation period for cocoa beans, no correlation was observed based on the mushroom family.

**Figure 3:**
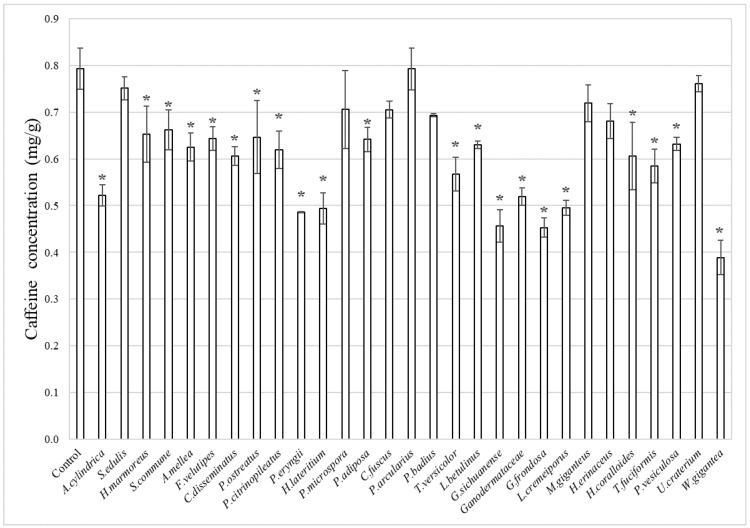
Caffeine concentration in fermented chocolate (mg/g). The error bars in the figure represent the standard deviation (n = 3). Asterisks (*) indicate significant differences compared with the control (*p* < 0.05).

Caffeine concentration in fermented chocolate was lower than the control for all mushrooms, except chocolate fermented with *P. arcularius* (Figure 3). Chocolate fermented with *P. arcularius* showed a caffeine concentration similar to that of the control. This suggests that fermentation with certain mushroom strains breaks down the caffeine in cocoa beans. In this study, compared to the control sample that underwent no fermentation, fermenting with mushrooms resulted in a decrease in caffeine content and a tendency toward increased theobromine content. This suggests that mushroom fermentation may have broken down the caffeine in the cacao beans and generated newly theobromine.

Theobromine and caffeine are types of alkaloids called methylxanthines, found in cocoa, tea leaves, coffee, and other plant sources. Caffeine and theobromine are closely linked in their respective biosynthetic pathways. In fungi and bacteria, the caffeine degradation pathway converts caffeine into theobromine, which is subsequently metabolized to 7-methylxanthine and finally to xanthine [25,26]. These steps involve sequential demethylation reactions catalyzed by specific demethylating enzymes. This suggests that mushrooms possess demethylating enzymes. We hypothesize that the activity of demethylating enzymes enables the conversion of caffeine into theobromine and 7-methylxanthine.

This finding supports a previous study that reported that when *Pleurotus ostreatus* fruiting bodies were cultivated on sawdust medium containing coffee grounds, and the mycelium was grown on potato dextrose agar supplemented with coffee grounds, the caffeine degradation products theobromine, 3-methylxanthine, and 7-methylxanthine were detected in both the fruiting bodies and the mycelium [27]. However, there are still no reports of *P. arcularius*, *P. vesiculosa*, or *U. craterium*—which are also mushrooms—degrading caffeine to produce theobromine, and the mechanism by which *P. arcularius*, *P. vesiculosa*, and *U. craterium* generate theobromine remains unknown.

Nevertheless, based on reports of caffeine degradation pathways and theobromine production in the same mushroom species, *P.ostreatus*, the reduction in caffeine concentration and the increase in theobromine concentration observed in chocolate fermented with mushrooms in this study were likely attributable to the presence of demethylating enzymes in the mushrooms *P. arcularius*, *P. vesiculosa*, and *U. craterium* possess demethylating enzymes. It is thought that during the cultivation period, these enzymes decomposed the caffeine contained in the cacao beans and generated theobromine.

In this study, the caffeine concentration of cocoa beans fermented with *P. arcularius* remained unchanged; however, the theobromine concentration increased. This suggests that theobromine was synthesized via a different pathway rather than being derived from the breakdown of caffeine. In the caffeine degradation pathway, demethylating enzymes convert caffeine into theobromine, 7-methylxanthine, and xanthine. The reverse reaction pathway occurs in the caffeine biosynthesis pathway: xanthine is converted to 7-methylxanthosine and then to 7-methylxanthine, ultimately forming theobromine (3,7-dimethylxanthine), which is then converted to caffeine (1,3,7-trimethylxanthine). In this process, methylation reactions occur in which methyl groups are added to the purine ring in the order N-7, N-3, and N-1 by the action of methyl transferase (N-methyltransferase), leading to the synthesis of theobromine and caffeine from xanthine [28].

If methylating enzyme (N-methyltransferases) are present in the mushroom along this pathway, it is conceivable that during the fermentation period of cacao beans, methyl groups could be added to the N-7 and N-3 positions of xanthine contained within the beans, enabling the production of theobromine. To date, there have been no reports of *P. arcularius* or other mushrooms possessing methyl transferase enzymes (N-methyltransferases). However, since the caffeine concentration in the cacao beans remained unchanged while the theobromine concentration increased, it was considered possible that *P. arcularius* possesses methyl transferase enzymes and newly produced theobromine from xanthine during the fermentation period of the cacao beans.

Therefore, another possibility is that the mushrooms possess both demethylase enzymes that break down caffeine and methylase enzymes that produce theobromine and caffeine from xanthine, both of which were active during the cultivation period.

There have been no reports of caffeine breakdown or theobromine production in *P. arcularius*, *P. vesiculosa*, or *U. craterium*, but the results of this study suggest the presence of demethylating and methylating enzymes, and further investigation of these enzymes is necessary to follow up on the results obtained in this study. However, by investigating and clarifying the mechanism of action of the enzymes, it may be possible to develop fermented chocolate with high theobromine and low caffeine concentrations through mushroom fermentation.

We focused on the species *P. arcularius*, *P. vesiculosa*, and *U. craterium*, which showed high theobromine concentrations, and conducted further analysis of their other components.

### 3.3. Production of Reducing Sugars Through Fermentation

Mushrooms possess various enzymes including amylase and cellulase. These enzymes are also active in fermented foods produced using mushroom mycelium where they increase the concentration of reducing sugars in the fermentation products [29]. We hypothesized that fermentation with mushrooms reduces the reducing sugar concentration in cocoa beans due to the amylase and cellulase present in the mushrooms.

**Figure 4:**
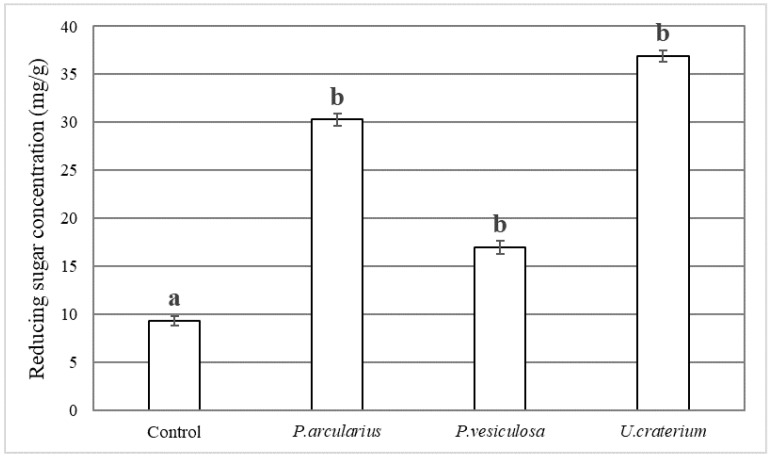
Reducing sugar concentration in fermented chocolate (mg/g). The error bars in the figure represent the standard deviation (n = 3). Different letters indicate *p* < 0.05 between groups.

Measurement of the reducing sugar concentration showed that the concentrations in beans fermented by *P. arcularius*, *P. vesiculosa*, and *U. craterium* were higher than those of the control (Figure 4). In particular, *U. craterium* had significantly higher reducing sugar concentration (36.9 mg/g) than the control (9.29 mg/g).

Cocoa beans contain carbohydrates, primarily dietary fiber, but also including lignin, hemicellulose, and cellulose, in that order [30]. On the other hand, many basidiomycetes are wood-decay fungi and possess hemicellulose and cellulase that degrade hemicellulose and cellulose [11]. *P. arcularius* belongs to the Basidiomycota phylum. It is classified as a white rot fungus and can decompose hemicellulose and cellulose [31]. On the other hand, *P. vesiculosa* and *U. craterium* belong to the Ascomycota phylum, are not classified as wood-decay fungi, and it has not been reported that they possess hemicellulose or cellulase. However, some ascomycetes behave as soft-rot fungi and decompose wood with high moisture content and have decompositional activity against cellulose [32]. This suggests that *P. vesiculosa* and *U. craterium* may also possess cellulolytic activity, despite the fact that Ascomycota are typically classified as wood-decay fungi that lack hemicellulase and cellulase.

In this study, fermentation using *P. arcularius*, *P. vesiculosa*, and *U. craterium* significantly increased the reducing sugar concentration compared with the control. This suggests that cellulose and hemicellulose in the cocoa beans were decomposed into reducing sugars, such as glucose, by cellulase and hemicellulose enzymes present in the mushrooms. Although fermentation with these species increases the concentration of theobromine, which contributes to bitterness, as the concentration of reducing sugars, which contribute to sweetness, also increased; therefore, the fermented chocolate may possess both bitterness and sweetness.

### 3.4. Production of Total Polyphenols by Fermentation

**Figure 5:**
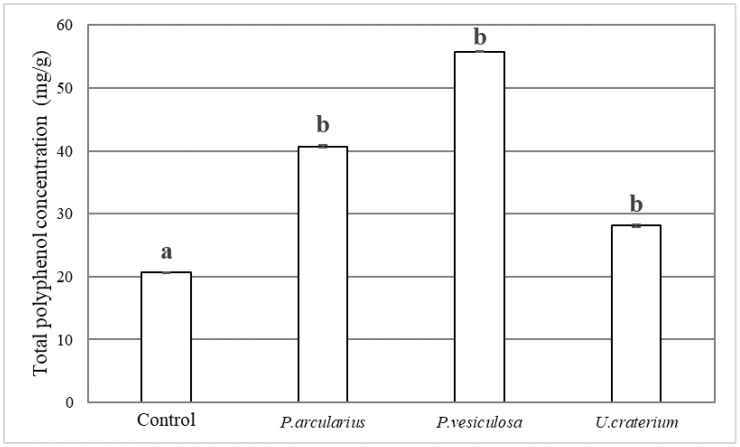
Total polyphenol concentration in fermented chocolate (mg/g). Total polyphenol concentration was quantified as epicatechin equivalents. The error bars in the figure represent the standard deviation (n = 3). Different letters indicate *p* < 0.05 between groups.

Chocolate fermented using mushrooms had higher total polyphenol concentrations than the control, with *P. arcularius* and *P. vesiculosa* in particular showing polyphenol concentrations more than twice that of the control (Figure 5). Cocoa beans are rich in lignin, a polymer of polyphenols, and polyphenols are released when lignin is decomposed.

*P. arcularius* is a white rot fungus capable of decomposing cellulose, hemicellulose, and lignin [11,31]. *P. vesiculosa* and *U. craterium* belong to the Ascomycota phylum. Some ascomycetes have been reported to decompose lignin [32]. These findings support this study as fermentation with *P. arcularius*, *P. vesiculosa*, and *U. craterium* decomposed the lignin in the cocoa beans, leading to an increase in the total polyphenol concentration.

### 3.5. Increased Antioxidant Activity Through Fermentation

**Figure 6:**
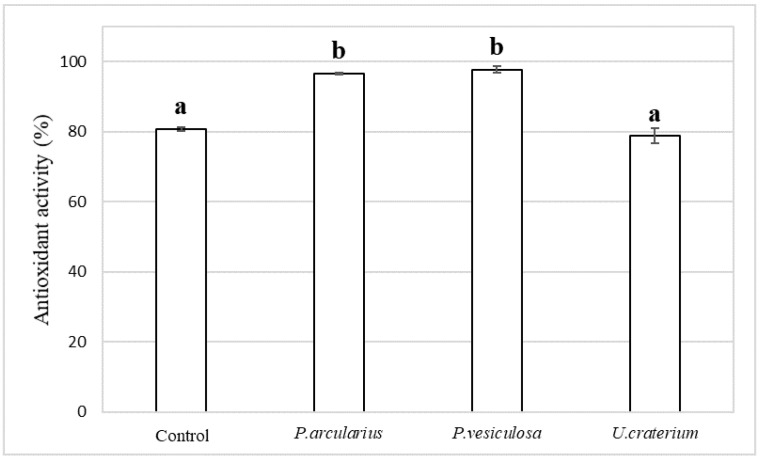
Antioxidant activity (%) in fermented chocolate. The error bars in the figure represent the standard deviation (n = 3). Different letters indicate *p* < 0.05 between groups.

The antioxidant activity measurement results showed higher values for *P. arcularius* and *P. vesiculosa*, which had high total polyphenol content, compared with the control (Figure 6). On the other hand, *U. craterium* showed antioxidant activity similar to that of the control.

The high antioxidant activity observed in chocolate fermented with *P. arcularius* and *P. vesiculosa* may be attributable to the release of antioxidant-active polyphenols associated with the increased total polyphenol concentration. On the other hand, antioxidant activity did not increase in chocolate fermented with *U. craterium*, despite its high total polyphenol content. This discrepancy may be explained by the presence of free polyphenols with inherently weak antioxidant activity or by oxidative degradation during the cultivation period, which may have diminished their antioxidant potential.

Polyphenols are compounds containing two or more phenol groups in a benzene ring. Polyphenols found in cacao beans include epicatechin and catechin, as well as procyanidin, which is a combination of epicatechin and catechin.

It has been reported that polyphenols contained in cacao beans exhibit higher antioxidant activity than black tea and green tea [33]. However, the antioxidant activity of each polyphenol, such as epicatechin and catechin, differs [34]. Therefore, even when the total polyphenol concentration is high, it may not directly lead to an increase in antioxidant activity.

The total polyphenol concentration measured in this study was not obtained by measuring the total polyphenol concentration in fermented chocolate using the Folin–Ciocalteu method; however, it was obtained by fractionating the obtained polyphenols and measuring each polyphenol individually. Furthermore, since the antioxidant activity was measured by determining the oxidation inhibition rate in fermented chocolate, the antioxidant effects of each polyphenol were not measured. Therefore, although lignin was decomposed in chocolate fermented with *U. craterium*, and the total polyphenol concentration increased, this may not lead to the further production of polyphenols that contribute to improved antioxidant activity.

Chocolate fermented with *U. craterium* did not exhibit increased antioxidant activity; however, its antioxidant activity was comparable to that of the non-fermented control, suggesting that mushroom fermentation did not diminish antioxidant capacity. Chocolate fermented with *P. arcularius*, *P. vesiculosa*, and *U. craterium* showed increases in theobromine concentration, reducing sugar content, and total polyphenol content; moreover, the antioxidant activity was similar to or higher than that of the control.

Collectively, these findings suggest that fermenting cacao beans with mushrooms enables the development of chocolate enriched in theobromine, reducing sugars, and polyphenols, with antioxidant activity that is maintained or enhanced compared with conventional chocolate.

## 4. Conclusions

This study investigated the use of mushrooms for the secondary fermentation of cocoa beans to further improve quality. When cocoa beans were fermented using 42 species of basidiomycetes and ascomycetes, mycelial growth was observed in 29 mushroom strains.

As a result of preparing 75% cocoa chocolate, the chocolate made with 17 strains showed higher theobromine concentrations compared to the control group, with chocolate fermented with *P. arcularius*, *P. vesiculosa*, and *U. craterium* showing significantly higher levels. Caffeine concentration was similar to or lower than the control in all mushroom strains. The increase in theobromine concentration and decrease in caffeine concentration may indicate theobromine synthesis from xanthine via methylation enzymes, or the generation of theobromine through the breakdown of caffeine by demethylation.

Furthermore, fermentation by *P. arcularius*, *P. vesiculosa*, and *U. craterium* increased the reducing sugar concentration and total polyphenol concentration. Fermentation by *P. arcularius* and *P. vesiculosa* showed excellent antioxidant activity.

Overall, our findings suggest that fermenting cacao beans with mushrooms produces chocolate that is richer in theobromine, reducing sugars, and polyphenols than conventional chocolate and has excellent antioxidant properties. However, for the efficient production of chocolate with high theobromine and low caffeine content through secondary fermentation, further research is required to characterize the methylation and demethylation enzyme in mushrooms.

## Figures and Tables

**Table 1:** Mycelium used in this study and cocoa bean fermentation period (days).

Scientific Name		Phylum	Class	Order	Family	Identification Number	Growth Medium	Culture Period (Days)
Genus	Specific Name							
*Agrocybe*	*cylindrica*	*Basidiomycota*	*Agaricomycetes*	*Agaricales*	*Bolbitaceae*	Ya-1 *	Matsutake Medium	33
*Sarcomyxa*	*edulis*	*Basidiomycota*	*Agaricomycetes*	*Agaricales*	*Sarcomyxaceae*	30526	PDA Medium	43
*Hypsizigus*	*marmoreus*	*Basidiomycota*	*Agaricomycetes*	*Agaricales*	*lyophyllaceae*	Bn-1 *	PDA Medium	71
*Schizophyllum*	*commune*	*Basidiomycota*	*Agaricomycetes*	*Agaricales*	*Schizophyllaceae*	4929	PDA Medium	47
*Armillaria*	*mellea*	*Basidiomycota*	*Agaricomycetes*	*Agaricales*	*Physalacriaceae*	7037	PDA Medium	55
*Flammulina*	*velutipes*	*Basidiomycota*	*Agaricomycetes*	*Agaricales*	*Physalacriaceae*	SA-1 *	PDA Medium	47
*Hymenopellis*	*radicata*	*Basidiomycota*	*Agaricomycetes*	*Agaricales*	*Physalacriaceae*	9785	PDA Medium	-
*Lentinula*	*edodes*	*Basidiomycota*	*Agaricomycetes*	*Agaricales*	*Omphalotaceae*	Sh-1 *	PDA Medium	-
*Coprinellus*	*disseminatus*	*Basidiomycota*	*Agaricomycetes*	*Agaricales*	*Psathyrellaceae*	7550	PDA Medium	54
*Agaricus*	*bisporus*	*Basidiomycota*	*Agaricomycetes*	*Agaricales*	*Agaricaceae*	7214	PDA Medium	-
*Calvatia*	*craniiformis*	*Basidiomycota*	*Agaricomycetes*	*Agaricales*	*Agaricaceae*	30530	PDA Medium	-
*Cyathus*	*stercoreus*	*Basidiomycota*	*Agaricomycetes*	*Agaricales*	*Agaricaceae*	9076	Matsutake Medium	-
*Pleurotus*	*ostreatus*	*Basidiomycota*	*Agaricomycetes*	*Agaricales*	*Pleurotaceae*	Hr-24 *	PDA Medium	49
*Pleurotus*	*citrinopileatus*	*Basidiomycota*	*Agaricomycetes*	*Agaricales*	*Pleurotaceae*	Ta-1 *	PDA Medium	47
*Pleurotus*	*eryngii*	*Basidiomycota*	*Agaricomycetes*	*Agaricales*	*Pleurotaceae*	Er-3 *	PDA Medium	54
*Pleurotus*	*abalonus*	*Basidiomycota*	*Agaricomycetes*	*Agaricales*	*Pleurotaceae*	Ka-1 *	PDA Medium	-
*Agrocybe*	*praecox*	*Basidiomycota*	*Agaricomycetes*	*Agaricales*	*Strophariaceae*	30258	PDA Medium	-
*Hypholoma*	*lateritium*	*Basidiomycota*	*Agaricomycetes*	*Agaricales*	*Strophariaceae*	W533 *	2% Malt Agar Medium	21
*Pholiota*	*microspora*	*Basidiomycota*	*Agaricomycetes*	*Agaricales*	*Strophariaceae*	Na-4 *	PDA Medium	40
*Pholiota*	*adiposa*	*Basidiomycota*	*Agaricomycetes*	*Agaricales*	*Strophariaceae*	Nm-1 *	PDA Medium	29
*Stropharia*	*rugosoannulata*	*Basidiomycota*	*Agaricomycetes*	*Agaricales*	*Strophariaceae*	30225	Matsutake Medium	-
*Auricularia*	*polytricha*	*Basidiomycota*	*Agaricomycetes*	*Auriculariales*	*Auricularaceae*	W9 *	2% Malt Agar Medium	-
*Cyclomyces*	*fuscus*	*Basidiomycota*	*Agaricomycetes*	*Hymenochaetales*	*Hymenochaetaceae*	9789	Matsutake Medium	21
*Climacodon*	*septentrionalis*	*Basidiomycota*	*Agaricomycetes*	*Polyporales*	*Irpicaceae*	W4 *	2% Malt Agar Medium	-
*Polyporus*	*arcularius*	*Basidiomycota*	*Agaricomycetes*	*Polyporales*	*Polyporaceae*	W122 *	2% Malt Agar Medium	41
*Polyporus*	*badius*	*Basidiomycota*	*Agaricomycetes*	*Polyporales*	*Polyporaceae*	30355	Matsutake Medium	35
*Trametes*	*versicolor*	*Basidiomycota*	*Agaricomycetes*	*Polyporales*	*Polyporaceae*	6516	PDA Medium	57
*Lenzites*	*betulinus*	*Basidiomycota*	*Agaricomycetes*	*Polyporales*	*Polyporaceae*	4963	PDA Medium	76
*Ganoderma*	*sichuanense*	*Basidiomycota*	*Agaricomycetes*	*Polyporales*	*Polyporaceae*	31863	PDA Medium	47
*Ganoderma*	*applanatum*	*Basidiomycota*	*Agaricomycetes*	*Polyporales*	*Polyporaceae*	Napa *	2% Malt Agar Medium	21
*Grifola*	*frondosa*	*Basidiomycota*	*Agaricomycetes*	*Polyporales*	*Polyporaceae*	Mi-h *	2% Malt Agar Medium	45
*Laetiporus*	*cremeiporus*	*Basidiomycota*	*Agaricomycetes*	*Polyporales*	*Laetiporaceae*	W8 *	2% Malt Agar Medium	28
*Meripilus*	*giganteus*	*Basidiomycota*	*Agaricomycetes*	*Polyporales*	*meripilaceae*	Tb-1 *	PDA Medium	41
*Sparassis*	*latifolia*	*Basidiomycota*	*Agaricomycetes*	*Polyporales*	*Sparassidaceae*	Hb-1 *	PDA Medium	-
*Hericium*	*erinaceus*	*Basidiomycota*	*Agaricomycetes*	*Russulales*	*Hericiaceae*	Ym-2 *	2% Malt Agar Medium	21
*Hericium*	*coralloides*	*Basidiomycota*	*Agaricomycetes*	*Russulales*	*Hericiaceae*	Sn-1 *	PDA Medium	33
*Tremella*	*fuciformis*	*Basidiomycota*	*Tremellomycetes*	*Tremellales*	*Tremellaceae*	8990	PDA Medium	43
*Morchella*	*esculenta*	*Ascomycota*	*Pezizomycetes*	*Pezizales*	*Morchellaceae*	33132	PDA Medium	-
*Peziza*	*vesiculosa*	*Ascomycota*	*Pezizomycetes*	*Pezizales*	*Pezizaceae*	30324	PDA Medium	34
*Urnula*	*craterium*	*Ascomycota*	*Pezizomycetes*	*Pezizales*	*Sarcosomataceae*	30137	PDA Medium	42
*Wynnea*	*gigantea*	*Ascomycota*	*Pezizomycetes*	*Pezizales*	*Sarcosomataceae*	8826	PDA Medium	48
*Cordyceps*	*militaris*	*Ascomycota*	*Sordariomycetes*	*Hypocreales*	*Cordycipitaceae*	5928	PDA Medium	-

- No mycelial growth was observed even after a cultivation period of 100 days or more. The numbers show NBRC No., * show Lab No. (A strain isolated by pure culture of wild-type bacteria in the laboratory).

## Data Availability

The original contributions presented in this study are included in the article. Further inquiries can be directed to the corresponding author.
